# Cases from Dr. Hamilton’s Clinic at the Buffalo Hospital of the Sisters of Charity

**Published:** 1859-01

**Authors:** J. Boardman


ART. II.—*Cases from Dr. Hamilton's Clinic at the Buffalo Hospital of the Sisters of Charity.* Reported by J. BOARDMAN, M. D.

*Hydrocele.* A. G., aged five weeks. General appearance healthy. Within one or two days after birth, his mother noticed that the left side of the scrotum was larger than the right. This swelling gradually increased, but she thought it had been stationary for the last week. She had not seen any swelling in the groin, and had just noticed this at the lower part of the scrotum. On examination, a tumor was found, without tenderness, pear shaped and fluctuating.

Dr. Hamilton punctured it with a narrow-bladed bistoury, and there escaped about ℥iss of a light straw-colored fluid. The child was ordered to be presented in about five weeks, but the mother has not appeared as yet.

T. S., aged 45 years, carpenter. About four years since, he received a blow upon the right side of his scrotum. Had had a little pain at times in that part; nine months since, noticed a slight swelling in the right side of the scrotum at the lower part; this had gradually increased, though accompanied with but very little pain. On examination, there was discovered a large, pear-shaped, fluctuating tumor, occupying the right side of the scrotum. The testicle could not be felt. There was no swelling in the groin.

Dr. Hamilton tapped it in front near the base, with a narrow bladed bistory, and let out about ℥x of a straw-colored fluid. He advised the patient to return when it filled, and to submit to a radical operation.

This disease is caused by the abnormal secretion of a serous fluid in the cavity of the tunica vaginalis. It may be congenital, the result of an injury, or of any cause exciting a chronic inflammation of this membrane. The fluid is secreted slowly and falls to the lowest part of the sac, gradually producing a swelling broad at the lower part, and becoming smaller as it approaches the body. The testicle is generally found at the upper and back side of the scrotum.

Hydrocele may be mistaken for fungoid disease of the testicle, hæmatocele, or hernia. But the diagnosis can generally be clearly made out by a careful investigation of the history of the case, noting the point where the swelling first appeared, its gradual increase, the comparative freedom from pain, the regularity of the surface of the tumor, its pyramidal or pear-shaped form, the general good health of the patient, and in most cases, by its semi-transparency; this latter sign is not always to be found; it is often wanting in old cases, where the tunica vaginalis has become thickened by inflammation, or the nature of the fluid changed so as to render the tumor opaque; but if present, it affords the surest means of diagnosis.

Congenital hydrocele will frequently, if left to itself, disappear,—the fluid being gradually absorbed. Sometimes the cure is effected by mild stimulating applications; or if the sac is punctured, it seldom returns. But in other cases, although simple tapping has effected a cure, it is looked upon as the exception, not the rule, and is now regarded by almost all surgeons as either a palliative, or preparatory operation. It is in this latter light esteemed by Dr. Hamilton, who advises tapping, at least once, before the performing of the radical operation. This is called a "safe operation," though Sir Astley Cooper relates cases he had seen where violent inflammation, and even death followed this "safe operation."

Various means have been at different times proposed to effect a radical cure: such as incision, excision of part of the sac, application of caustic, the seton, injections of some irritant fluid into the tunica vaginalis, etc., all having one end in view, the exciting of inflammation and the production of granulation and adhesion. But the inflammation is not always under control: at times the life of the patient is endangered. Of late years, surgeons have generally used either injections, or incisions. This last, is Dr. Hamilton's favorite method. First, as in the case above-mentioned, he advises simple tapping, and when the sac again fills, he would lay it open by means of a free incision, placing a piece of lint in the wound, and immediately applying a poultice to the parts, keeping the patient in bed and snpporting the scrotum with pads of soft cotton. He thinks that the inflammation is less severe, if the patient has been once tapped before the radical operation is performed; also, in his own hands, he can control the inflammation excited by a free incision and a piece of lint, with much greater ease than that excited by irritating injections.

*Hare-lip.* Dr. Hamilton brought before the class, B., aged 13 years, having congenital hare-lip.

On examination, a single fissure of the upper lip was found, a little to the left of the median line, extending to the base of the nose. The lip had never been operated upon. Neither the father, mother, or any of her family have had hare-lip.

Dr. Hamilton operated, by cutting a narrow strip from both sides of the fissure, with a strong pair of scissors; he then dissected the left ala of the nose from the superior maxillary bone. No ligature was placed upon the superior coronary artery, for the bleeding would cease, he said, when the fissure was closed. A needle armed with silk, was placed through the entire thickness of the lip, upon the one side of the fissure, and brought out on the other, in the line of union of the skin and the red part, about one-quarter of an inch from the raw surface. Another suture was introduced midway in the fissure, and a third, at the upper angle. The lower of these sutures was first tied, and then the others, an assistant standing behind the patient, and bringing the parts together, by a hand placed on each side of the face, pushing the cheeks forward. These sutures, except sometimes the upper one, he passes through the entire thickness of the lip, and at one-quarter of an inch from the edge of the cut surface. Dr. Hamilton then applied two pieces of adhesive plaster, shaped like a dumb-bell, extending from ear to ear, in such a manner as to take off all strain from the sutures. The third day, the lip was dressed before the class, an assistant standing behind the patient, with both hands supporting, and bringing forward the cheeks. Dr. H. then cut the plaster, each side of the nose, and carefully removed it; the wound was well cleaned, and as the parts seemed united, the upper suture was removed, and the lip was again dressed with adhesive plaster, in the same manner as at first. The other sutures were removed the fifth day The plasters were used till the sixteenth day, when the patient was dismissed, cured.

——, aged 18 years, with a single congenital fissure of the upper lip, which had been unsuccessfully operated upon; Prof. Hamilton operated and dressed it in the same manner as above described. The upper suture was removed the fourth day, and the others on the fifth and sixth days. The plasters were removed, and the patient was dismissed, with a perfect lip, on the fourteenth day.

Dr. Hamilton said, hare-lip was divided into three principal varieties; single, double, and complicated.

Single, when the lip was divided by but one fissure.

Double, if two fissures existed.

Complicated, if the fissures extended to the superior maxillary, or palate bone.

This is one of the most common of congenital deformities, rarely, if ever, occurring, exactly in the centre of the lip, but a little to the right or left of the median line.

In examining a number of cases in Dr. Hamilton's notes, for the cause, I find that in more than one-fourth of all the cases, one, or both, of the parents had a short upper lip. Many of the mothers gave as a reason, the drawing of a tooth while they were in the family way. Two, or three, to having seen persons with hare-lip, etc.

There is great difference of opinion amongst surgeons, as to the time most desirable for the operation; some advising to operate within a few hours, or days, after birth, and others recommending a delay of from two to eight years.

"Ten days is said to be the youngest age at which the operation of hare-lip has been performed at King's College Hospital, London."

"Dr. Friedburg relates three cases of hair-lip operation performed at the following ages, viz: Fourteen hours, ten hours, and three hours after birth. Chloroform was administered in each instance, and they all terminated favorably. The same writer advocates the early operation for hare-lip, in his recent work."

"Dr. Dawson (Dub. Med. Press, 1842) operated on a child having a simple hare-lip seven hours after birth. The pins were removed in forty-eight hours, and in two days more, the union was so perfect, that the mother, who had not seen her child, did not believe any deformity had existed. Mr. Bateman operated four hours after birth, where there was also fissure of the palate, so great as to admit the mother's thumb. The operation succeeded, and the fissure of the palate contracted, so as scarcely to admit the edge of a sheet of writing-paper. Malgaigne advocates early operation, and has operated nine hours after birth. P. Dubois and Guersant prefer operating immediately after birth."—*N. Y. Journal of Medicine, Nov.*, 1857.

Sir Astley Cooper writes: "It is undoubtedly true that adhesion is most sure to be lasting after the period of dentition, and that this operation, therefore, scarcely ever fails when performed between two years and adult age; on the contrary, during dentition it is attended with some danger. * * * Soon after birth, the operation often fails; the danger, however, is much greater; the nervous system is then so exceedingly irritable, that convulsions are easily produced, and the loss of a small quantity of blood occasions a fatal influence. * * * The conclusions, therefore, as far as my own experience dictates, are these: That prior to six months, there is danger of a want of union, and even of the loss of life; that from six months to two years, during the period of dentition, the operation should not be performed; that after dentition is completed, there is little risk of failure, either as regards the union of the lip, or the life of the child. In those cases in which a fissure has existed in the upper jaw, the union of the upper lip has, by its pressure upon the bone, led to an approximation of the edges of the fissure, so as to produce considerable advantage by the early operation."—*The Princ. and Prac. of Surgery, by Sir A. Cooper. Ed. by A. Lee, London*, 1836.

Dr. Hamilton has operated once with hare-lip pins, (his first case,) and *forty-one times* with the interrupted suture, notes of which I have seen and examined. In only three of his cases has the lip again opened. One of them was torn open by accident, and the other two (one iu which the hare-lip pin was used) passed from his care immediately after the operation, and he did not, on that account, consider them fair results. in all the other cases, union of the lip took place, and did not tear open. He attributed his success to the great care he used in dressing; in constantly, by means of adhesive plaster, keeping all strain from the sutures; and also, the use of his interrupted sutures. Ten days from birth is his earliest operation. Of his forty-two cases, seven have died.

| | | | | | | |
|---|---|---|---|---|---|---|
| One operated on in | June, | four | weeks | after birth, | died in | two months. |
| " " " | April, | eight | " | " | " | ten days. |
| " " " | June | " | " | " | " | three months. |
| " " " | August, | five | months | " | " | six weeks. |
| " " " | Sept., | six | " | " | " | five " |
| " " " | Oct., | seven | " | " | " | ten days. |
| " " " | June, | nine | " | " | " | four weeks. |
| " " " | Aug., | twelve | " | " | " | eight days. |

The conclusions that he draws from his own experience, are these: That the danger to the life of the patient is inversely to the age; that the operation is more successful, leaving less deformity, the earlier it is performed; that the operation should not be performed during the period of dentition (to which all surgeons, I believe, agree); nor during the hot months of summer, if it can be avoided.

In all of his own cases, death took place by means of diarrhœa, or cholera infantum. He thinks that this operation in children is more frequently followed by diarrhœa, than almost any other; and one cause, is the swallowing of blood during the operation, which, in a young child, it is almost impossible to prevent; therefore it is especially necessary to avoid the hot months.

Chelius, in his surgery, (edited by South, and again edited by G. W. Norris,) writes: "Hare-lip can only be cured by operation. Although experience has shown that the operation may be successful in very young children, it is, however, best to delay it till eight months. Only when wolf's jaw is connected with hare-lip, and the child cannot suck, may the operation be undertaken within the first six months. In children of two years, the operation may be delayed till they become intelligent."

In a note, the opinion of Lawrence is given, "who recommends the operation as early as the third, fourth, or fifth months; and of Mütter, who operates upon children three, four, and five days old. Mr. South would never perform it under two years, and he prefers the sixth, or eighth year."

The operation consists in making raw the edges of the fissures, and binding the edges together. This is done in various ways.

Dr. Zadoc Howe, in the Amer. Jour. Med. Science, vol. 7, 1830, describes his mode of operating: which consisted in making the edge of the fissure concave, like this, ( ), so that when brought together, there might be no tendency to a notch at the lower edge of the lip. He used one interrupted suture, and one gold pin, a little curved, with a steel point, withdrawing the pin in about sixty hours. He used adhesive plaster, extending from cheek to cheek.

The late Dr. Homer, of Philadelphia, introduced a piece of wood under the lip, and with a bistoury cutting upon it, curved both sides of the fissure. Introducing two hare-lip pins, made of silver, with steel points, which could be removed, half through the thickness of the lip, twisting a ligature in the form of a figure of 8 over the ends, and dressing with a narrow strip of plaster.

Sir Astley Cooper tells us that "Mr. Cline, who had great experience in his profession, preferred, and in his lectures, recommended, the interrupted suture." He himself says, "that it may be very successfully performed with either; but the interrupted suture is the most simple, and, as far as I have seen, equally effectual; it has this great advantage, that it prevents the disturbance to the adhesion, which the lip receives in the removal of the pins." He writes, "there is not any necessity for applying adhesive plasters." He removed, as a general rule, the upper suture on the fourth day, and the lower one upon the fifth day. In double hare-lip, he operated upon only one fissure at a time.

Dr. Hamilton's mode has been described. He depends much upon the use of plasters, renewing them as often as they become the least loose; he makes but one operation, even in cases of double hare-lip.

Ambrose Raze, who lived in the sixteenth century, "was the first to use the twisted suture in hare-lips, copying the mode of application from the manner in which the ladies and tailors of the day wound the thread around the needle."—*Miller's Princ. of Surg.*

The hare-lip. pins are mostly used in this country; but Dr. Hamilton thinks they are much inferior to the interrupted suture, because, not passing through the entire lip, they do not prevent the inner line of the lip from separating, thus leaving but a part of the thickness of the lip to unite. The ends are liable, especially in infants, to be hit or caught upon the clothing, and thus torn out; they allow but a very narrow strip of plaster to be used; and lastly, it often happens, that in removing the pins, the lip tears open, while the interrupted suture is free from all of these objections. Surely his forty-two cases are a strong argument in favor of the interrupted suture.

